# Sparse Subsystem Discovery for Intelligent Sensor Networks

**DOI:** 10.3390/s26010288

**Published:** 2026-01-02

**Authors:** Heli Sun, Xuechun Liu, Miaomiao Sun, Ruichen Cao, Bin Xing, Liang He, Hui He

**Affiliations:** 1 State Key Laboratory of Communication Content Cognition, Beijing 100733, China; 2 Faculty of Electronic and Information Engineering, School of Computer Science and Technology, Xi’an Jiaotong University, Xi’an 710049, China

**Keywords:** sparse subsystem discovery, reinforcement learning, graph neural network

## Abstract

The Sparse Subgraph Finding (SGF) problem addresses the challenge of identifying sub–graphs with weak social interactions and sparse connections within a graph, which can be effectively modeled as discovering sparse subsystems in intelligent sensor networks. Traditional methods often rely on manually designed heuristics, which are computationally expensive and lack scalability, especially when dealing with complex sensor network systems. In this paper, we propose RL-SGF, a novel framework that integrates deep reinforcement learning and graph embedding through joint optimization to overcome these limitations. By simultaneously optimizing subsystem sparsity and representation learning within a unified framework, RL-SGF enhances both the effectiveness and robustness of the model in sensor network applications. Experimental results on synthetic and real-world datasets, including social networks, citation networks, and sensor network simulations, demonstrate that RL-SGF outperforms existing algorithms in terms of efficiency and solution quality, making it highly applicable to real-world sparse subsystem discovery scenarios in intelligent sensor networks.

## 1. Introduction

The widespread use of graph data in fields such as social networks, citation networks, biological networks, and intelligent sensor network systems has established graph mining as a critical area in recent research [1,2,3,4,5]. Among its tasks, the Sparse Subgraph Finding (SGF) problem is notable for identifying weakly connected subgraphs within networks [6,7], which correspond to sparse subsystems in complex sensor networks. These sparse sub– systems play a critical role in understanding network structures and support applications such as group formation, fraud detection, network reliability analysis, and fault tolerance in sensor networks [8,9,10]. For example, in peer review processes for academic journals and conferences, SGF techniques can help assign reviewers with relevant expertise while avoiding conflicts of interest, thereby ensuring the integrity of the review process. Similarly, in intelligent sensor networks, identifying sparse subsystems enables efficient resource allocation, anomaly detection, and system robustness enhancement.

SGF is a typical Graph Combinatorial Optimization (GCO) problem, characterized by its NP-hardness and high computational complexity [11,12,13]. Solving it often requires optimizing sparse metrics (e.g., k-triangles and k-lines) under specific constraints [10,14,15], which can be interpreted as evaluating connectivity sparsity in sensor network subsystems. Traditional approaches rely heavily on heuristic or meta-heuristic algorithms, such as Genetic Algorithms (GA), Simulated Annealing (SA), and Particle Swarm Optimization (PSO). These methods, inspired by natural processes, demonstrate strengths in global optimization tasks. For instance, He et al. [16] proposed a memory-based genetic algorithm that significantly improved subgraph coverage efficiency. However, in most cases, these methods face significant limitations in computational efficiency and scalability, particularly when applied to large-scale sensor network systems. Metaheuristic algorithms typically require numerous iterations to explore the global search space, resulting in prohibitive computational costs for large-scale networks [17,18]. This necessitates the design of specialized optimization strategies for execution efficiency and computational time, particularly in terms of technical and hardware optimization [19]. Additionally, the performance of these methods is highly sensitive to parameters (e.g., population size, mutation rate), and the parameter-tuning process often requires extensive experimentation, which limits their practical flexibility in dynamic sensor network environments [20].

Deep Reinforcement Learning (DRL), in contrast, offers a promising alternative for solving graph-related optimization problems in sensor networks. Combining deep learning’s representation capabilities with reinforcement learning’s decision-making power, DRL enables adaptive optimization strategies for graph-related objectives [2,3,4,5,6,7,8,9,10,11,12,13,14,15,16,17,18,19,20,21,22,23,24]. By interacting with the graph environment, DRL iteratively learns optimization strategies, effectively capturing complex patterns and dependencies that traditional methods often fail to model. However, traditional deep subgraph mining methods typically treat graph embedding learning and subgraph optimization as separate steps [25]. Models first learn graph embeddings through neural networks and subsequently use them for subgraph detection.

This decoupled approach can create mismatches between the learned embeddings and the subgraph mining task, particularly in complex sensor network systems where subsystem sparsity is critical [26]. 

In sensor-network applications, identifying sparse subsystems is closely related to energy efficiency, robust subsystem formation, and fault detection. Prio works have tackled distributed detection of sparse signals under energy constraints [26], energy-efficient sensory data collection leveraging spatiotemporal correlations [27], and connectivity-optimized topologies for directional sensor deployments [28]. These studies motivate the use of sparsity-aware subgraph discovery in sensor networks. RL-SGF addresses these practical needs by finding compact k-node subsystems that reduce communication 66 and improve detection reliability while explicitly optimizing the PF metric.

To overcome the limitations of traditional methods, including computational inefficiency, lack of scalability, and decoupled optimization, this paper proposes RL-SGF, a novel framework designed to address the SGF problem in the context of intelligent sensor networks. RL-SGF leverages the strengths of DRL to overcome the iteration-heavy nature and parameter sensitivity of heuristic and meta-heuristic algorithms for large-scale networks. Moreover, RL-SGF integrates graph embedding learning and sparse subsystem optimization into a unified framework. Unlike traditional methods that treat training and optimization as separate processes, RL-SGF employs a joint optimization strategy, directly incorporating sparse metrics into the optimization process. This approach enables a seamless integration of embedding representations with sparse subsystem discovery objectives, reducing error propagation between modules and significantly improving performance in sensor network applications.

Specifically, RL-SGF uses DRL’s representation learning and strategy optimization capabilities to dynamically adjust optimization paths through interactions with the graph environment. This allows the model to capture complex graph patterns and dependencies effectively while substantially reducing computational costs. Additionally, RL-SGF not only identifies weakly connected subgraphs but also optimizes them efficiently, striking a balance between computational efficiency and solution quality for sparse subsystem discovery in sensor networks. Experiments on both synthetic and real-world datasets, including sensor network simulations, demonstrate that RL-SGF outperforms existing methods in terms of both solution quality and computational efficiency.

## 2. Related Work

### 2.1. Sparse Subgraph Finding (SGF) Methods

The SGF task originates from early work on finding tenuous groups, which aims to identify node sets with weak social interaction patterns [10]. The Sparse Subgraph Finding (SGF) problem, a type of Graph Combinatorial Optimization (GCO), has garnered significant attention in recent years. Traditional SGF methods often use heuristic algorithms to optimize subgraph structures based on metrics like k-triangle and k-line [6,7,10,14,15]. These metrics assess connection density, aiming to identify subgraphs with weak or sparse connections. Compared with these heuristic SGF formulations that depend on predefined structural metrics such as k-triangle and k-line, RL-SGF learns a policy explicitly optimized for PF minimization, enabling it to detect sparse connectivity patterns that are not captured by fixed handcrafted rules.

One of the seminal works in this area is the TERA algorithm proposed by Shen et al. [14,15], which focuses on minimizing k-triangles to identify sparse subgraphs. The TERA algorithm traverses the graph, removing nodes and their associated edges to reduce the number of k-triangles, thereby isolating subgraphs with minimal internal connections. Similarly, the WK algorithm by Li et al. [10] uses a max-heap to identify and remove nodes forming k-lines, reducing their count within the subgraph. While these methods have been effective in certain contexts, they are often computationally expensive and struggle to scale with larger networks. *n* contrast to TERA and WK, which rely on deterministic pruning strategies driven by structural statistics, RL-SGF performs end-to-end learning with dynamic graph embeddings, allowing the agent to identify sparsity-inducing nodes through data-driven policy optimization rather than fixed removal operations.

Another key limitation of traditional SGF methods is their reliance on predefined heuristics, which may not fully capture the complex and dynamic relationships inherent in real-world social networks [29]. This has led to the exploration of more advanced techniques that can better adapt to the evolving nature of these networks and provide more robust solutions. Different from these heuristic-based SGF approaches that struggle to model complex and evolving network relationships, RL-SGF unifies representation learning and decision optimization, enabling adaptive sparsity discovery under diverse and dynamic graph topologies. Recent advances in subgraph-level representation learning emphasize that learning representations at the subgraph (or motif) granularity can markedly improve down- stream tasks that depend on higher-order structural patterns. Representative works include SubGNN [30], which proposes a subgraph routing mechanism to capture both internal topology and external positional context of subgraphs, motif-based convolution modules that incorporate motif vocabularies into embedding learning [31], and comprehensive surveys on graph pooling methods that summarize hierarchical and flat pooling strategies [32]. These approaches provide structural priors and pooling mechanisms useful for identifying sparse structural motifs. Compared to these methods, RL-SGF performs end-to-end joint optimization: node/subgraph embeddings are updated within the reinforcement learning loop so that representations are directly aligned with the PF minimization objective.

### 2.2. Deep Learning and Reinforcement Learning in GCO 

The integration of Deep Learning (DL) and Reinforcement Learning (RL) has provided promising solutions to Graph Combinatorial Optimization (GCO) problems, such as Sparse Subgraph Finding (SGF) [33,34,35]. These approaches utilize advanced neural networks to automatically derive graph representations, capturing complex patterns and relationships that traditional methods may fail to detect [33]. Specifically, Deep Reinforcement Learning (DRL), by combining the strengths of both DL and RL, has emerged as a powerful technique for solving intricate graph optimization challenges, demonstrating substantial improvements in computational efficiency and solution quality [33,35]. DRL frameworks like GCO-DQN [34] leverage graph neural networks to represent entities in the network, enabling more effective decision-making for complex tasks like team formation and community detection. Compared with these DRL-based GCO frameworks that target general combinatorial objectives, RL-SGF integrates sparsity-aware embedding updates into every decision step, enabling the policy to be optimized directly for PF minimization rather than for task-agnostic rewards. A recent survey by Tam et al. provides a comprehensive overview of the integration of GNNs and DRL in intelligent end-to-end networking, highlighting the rapid growth and increasing significance of graph-based reinforcement learning frameworks [36]. Wei et al. combine graph-based topological embedding with DRL for autonomous voltage control, reinforcing the importance of joint representation learning and decision optimization in graph-based environments [37]. Elias et al. [37] introduced a comprehensive framework that combines reinforcement learning with graph embedding techniques to address classical GCO problems such as the Minimum Vertex Cover Problem (MVCP) and the Traveling Salesman Problem (TSP). This approach uses a neural network to learn graph embeddings, enabling the RL agent to optimize combinatorial objectives. Similarly, Li et al. [38] integrated Graph Convolutional Networks (GCNs) with heuristic algorithms to solve various GCO problems, demonstrating significant improvements in both computational efficiency and solution quality. Wang and Liang demonstrate that combining GNNs with self-attention and reinforcement learning significantly improves optimization performance in complex networked systems such as supply chain routing. Their findings highlight the importance of joint graph representation and policy learning, which is consistent with the motivation of RL-SGF [39]. Zhao and Yan apply deep reinforcement learning with graph convolution to dynamic on-street parking navigation, showing that RL-driven decisions are highly effective in real-world, large-scale graph environments [40].” While these approaches highlight the strengths of jointly leveraging GNNs and DRL for classical graph optimization tasks, RL-SGF differs by tightly coupling sparsity-aware representation learning with PF-oriented policy optimization in an end-to-end manner specifically designed for the SGF problem.

In our previous work, we introduced GNNM [29], which, to the best of our knowledge, was the first application of Graph Neural Networks (GNNs) to the SGF problem. This work demonstrated the ability of neural networks to effectively capture the sparsity of subgraphs, establishing a solid foundation for further exploration of DRL techniques in this field. While GNNM shows significant promise in improving the scalability and efficiency of SGF methods, it also proves effective in identifying subgraphs that traditional heuristic-based approaches might fail to detect. Recent motif-aware GNN approaches demonstrate that deep models can effectively capture sparse structural patterns, further motivating the integration of graph neural networks into SGF [29]. Unlike these applications of GNN-enhanced RL in routing or real-time control tasks, RL-SGF focuses on sparsity-driven k-node subgraph extraction and explicitly optimizes the PF objective under strict node-budget constraints, constituting a fundamentally different combinatorial setting. Version 14 December 2025 submitted to Journal Not Specified 5 of 28 Recent research has begun to apply reinforcement learning to graph sparsification and structure-editing tasks. For example, SparRL [41] presents a DRL-based framework to remove edges while preserving chosen graph properties, and other RL-driven structure editing methods learn topology modification policies optimized for downstream objectives. These frameworks show that RL can flexibly adapt sparsification goals beyond classic sampling schemes. RL-SGF differs from graph-editing RL in that it performs k-node subgraph selection under the PF objective rather than editing the global graph; the agent selects a compact subsystem instead of changing the entire topology. Although several DRL–GNN frameworks, such as GCO-DQN, have demonstrated the effectiveness of jointly learning graph representations and decision policies for general graph combinatorial optimization problems (e.g., minimum vertex cover or routing), their optimization objectives are fundamentally different from Sparse Subgraph Finding (SGF). Specifically, these methods aim to optimize task-agnostic combinatorial objectives and do not explicitly model sparsity-oriented subgraph metrics such as PF, k-line, or k-triangle. In contrast, RL-SGF is specifically designed for k-node sparse subgraph extraction, where sparsity is directly encoded into both the reward function and the embedding update process. Therefore, RL-SGF differs from existing DRL–GNN pipelines not in the general idea of joint learning, but in its task-specific formulation and sparsity-driven optimization objective.

### 2.3. Dynamic and Spectral Graph Sparsification

Dynamic (temporal) graph sparsification refers to algorithms that maintain a sparse representation of a graph under edge insertions and deletions, accommodating evolving structures in streaming settings [42]. Spectral graph sparsification focuses on approxi- mating the original graph with a sparsifier that preserves key spectral properties (e.g., Laplacian eigenvalues) and hence the global behavior of graph algorithms [43]. Recent works such as dy GRASS propose efficient dynamic spectral sparsifiers via localized random walks and parallel computation to support both incremental and decremental updates [42]. Spectral sparsification approaches often rely on matrix computations and spectral approximations, which differ from the subgraph extraction objective. In contrast, RL-SGFtargets compact k-node subsystem extraction under the PF objective without requiring full spectral decomposition, making it more suitable for time-sensitive and resource-constrained sensor-network environments.

### 2.4. Optimization and Scalable Graph Mining

Mixed-Integer Linear Programming (MILP) and Integer Linear Programming (ILP) based formulations have been extensively used for exact or approximate graph optimization and combinatorial problems, though they generally do not scale to large networks due to NP-hardness [44]. Meanwhile, scalable graph mining techniques such as graph sampling, compression, and distributed processing reduce computational and memory burdens on large graphs and support efficient neural network training [44]. These methods either provide optimal solutions at high computational cost or trade optimality for efficiency via divide-and-conquer and sampling strategies. Compared with optimization-based or scalable mining frameworks, RL-SGF offers a data-driven policy that adaptively learns sparse subgraph extraction strategies without exhaustive optimization or global preprocessing, balancing solution quality and scalability.

## 3. Problem Formulation

In this section, we present the formal definition of the Sparse Subgraph Finding (SGF) problem, accompanied by an analysis of its computational complexity and the evaluation metrics employed. We particularly emphasize the Potential Friends (PF) metric, which plays a critical role in the RL-SGF model’s approach to solving the SGF problem.

### 3.1. Graph Representation

Consider an undirected graph *G* = (*V*, *E*), where *V* is the set of vertices and E is the set of edges connecting these vertices. In the context of social networks, nodes *v* ∈ *V* often represent individuals, and edges *e* ∈ *E* signify the relationships or interactions between them. The graph can be represented by an adjacency matrix *A*, where *A_ij_* = 0 indicates no direct connection.

### 3.2. Sparse Subgraph

A sparse subgraph *T* ⊆ *G* is defined as a subgraph where the connections between nodes are minimal or weak. Formally, a subgraph *T* = (*VT*, *ET*) is considered sparse if the relationships within *VT* ⊆ *V* and *ET* ⊆ *E* exhibit low density or sparse connectivity. Metrics such as *k*-triangle and *k*-line are commonly employed to evaluate the sparsity of a subgraph, where fewer triangles or lines indicate greater sparsity.

### 3.3. Sparse Subgraph Finding (SGF) Problem

The SGF problem can be formally defined as follows: Given an undirected graph *G* = (*V*, *E*) andapositive integer *k*, the objective is to identify a subgraph *T* ⊆ *G* such that *T* contains exactly *k* nodes and minimizes a chosen sparse metric, such as the number of *k-triangles* or *k-lines*.

Problem Definition:

- Input:
  - An undirected graph *G* = (*V*, *E*).
  - A positive integer *k*, representing the size constraint for the subgraph.
- Output:
  - A subgraph *T* = (*VT*, *ET*) where *VT* ⊆ *V*, |*VT*| = *k*, and *T* minimizes the sparse metric.

### 3.4. Complexity Discussion

Given the NP-hard nature of the SGF problem, solving it poses significant computational challenges, particularly as the size of the input graph increases. The problem’s inherent inapproximability makes it infeasible to find an exact solution within a reasonable time frame for large-scale graphs using traditional methods. This computational complexity is further exacerbated by the need to evaluate multiple sparse metrics, such as *k*-triangles and *k*-lines, across various subgraphs, each requiring substantial computational resources. Consequently, developing efficient algorithms capable of approximating optimal solutions in a scalable manner is critical for advancing the study of SGF.

### 3.5. Evaluation Metrics

To evaluate the quality of the identified sparse subgraph, several metrics can be used:

- *k*-Line Metric: This metric calculates the number of *k-lines* (paths of length *k*) within the subgraph. Fewer *k-lines* suggest weaker connectivity [10].
- *k*-Triangle Metric: This metric counts the number of triangles (three nodes where all pairs are connected) in the subgraph. Fewer triangles indicate greater sparsity [14,15].
- Potential Friends (PF) Metric: An advanced metric introduced in recent research, PF combines the *k*-triangle and *k*-line metrics to more effectively capture latent relation-ships between nodes, enhancing the assessment of subgraph sparsity [29].

## 4. Method Design of RL-SGF

In this section, we provide a comprehensive overview of the RL-SGF framework, detailing how we integrate deep reinforcement learning with graph embedding techniques to address the Sparse Subgraph Finding (SGF) problem. We also introduce key definitions relevant to the application of deep reinforcement learning in this context. Our approach overcomes the limitations of traditional heuristic methods by enabling the model to automatically learn subgraph structures that effectively minimize social interactions within large-scale networks. The section concludes with an analysis of the model’s time complexity, including a comparative evaluation against traditional methods.

### 4.1. Graph Embedding Module

The graph embedding module is a critical part of the RL-SGF framework, responsible for transforming the graph’s structural information into a lower-dimensional space that can be efficiently processed by the reinforcement learning agent. Similarly to recent work in graph-based control systems, Wei et al. show that incorporating topological graph embeddings is essential for effective DRL-based decision-making in complex networks, which supports the design of our embedding module [37].Unlike traditional methods that rely on predefined features, this module dynamically learns the representations of nodes based on their relationships and the surrounding graph structure. We employ a sampling aggregation technique to reduce computational complexity while preserving essential connectivity patterns within the graph. Specifically, for each node *v*, a fixed-size set of neighboring nodes is sampled. The embedding for each node *v* is then updated as:

(1) $$h_{v}^{\left(i\right)}=ReLU\left(a_{1}\cdot Concat\left(h_{N(v)}^{\left(i-1\right)},h_{v}^{\left(i-1\right)}\right)\right)+a_{2}\cdot x_{v}$$

where $h_{v}^{\left(i-1\right)}$ represents the embedding of node v from the previous iteration, $h_{N(v)}^{\left(i-1\right)}$ denotes the aggregated embeddings from the neighbors of $v,\;a_{1\;}and\;a_{2}$ are learnable parameters used to update the node embeddings. They control the weights of the information from neighboring node embeddings and the current node embedding during the update process. These two parameters are automatically adjusted through backpropagation during the training process to optimize the performance of the entire graph embedding model. They are set during model initialization and are learned through error backpropagation in subsequent gradient descent steps. and $x_{v}$ is a Boolean variable indicating whether node v is part of the feasible solution. *A* feasible solution is defined as a node set $V_{T}$ that satisfies the node count constraint $\sum_{v\in V}x_{v}=k$ and minimizes the sparse metric (e.g., the PF metric). During training, $x_{v}$ is dynamically updated, and the model ensures feasibility by checking the constraint $\sum_{v\in V}x_{v}=k$ in real-time. If the condition is violated, the solution is penalized in the reward function, guiding the model toward feasible and optimal subgraphs.

The embedding module is trained in an unsupervised manner using a loss function that ensures nodes with similar topological distances have similar embeddings, while those with distant topological distances have distinct embeddings:

(2) $$L_{embed\;}=\;\log\left(1-\sigma \left(h_{i}^{T}h_{j}\right)\right)+E_{v_{n}\sim p_{n\left(v_{i}\right)}\log\left(\sigma \left(h_{i}^{T}h_{v_{n}}\right)\right)}$$

Here, σ(·) is the sigmoid function, and $p_{n}\left(v_{i}\right)$ denotes the negative sampling distribution for non-neighbor nodes. For each target node $v_{i}$, a fixed number of negative samples $v_{n}$ are drawn uniformly from the set of non-adjacent nodes. These samples are used to compute the loss in (2), encouraging representation differences between *v_i_* and negative samples, while promoting similarity between *v_i_* and its immediate neighbors. This strategy enhances the expressiveness of the learned embeddings and captures key structural properties of the graph.

### 4.2. Deep Reinforcement Learning Module

This subsection presents the design and implementation of the Deep Reinforcement Learning (DRL) module in the RL-SGF model for solving the sparse Subgraph Finding (SGF) problem. By modeling states and actions, incorporating the Potential Friends (PF) metric for sparse measurement, defining a tailored reward function, and utilizing a Deep Q-Network(DQN), the RL-SGF framework effectively reformulates the subgraph discovery process as a sequential decision-making problem. This enables robust optimization for constructing weakly connected subgraphs. The effectiveness of DRL-based node selection has also been validated in recent work such as GAPO, where Zhao et al. employ graph attention and reinforcement learning to guide task offloading decisions in multi-hop vehicular networks [45].

#### 4.2.1. States and Actions in RL-SGF

The definitions of states and actions are central to the DRL module, as they describe how the agent perceives the environment and selects nodes to construct the subgraph. At time step *t*, the state *S_t_* includes the remaining graph $G_{t}=\;\left(V_{t},E_{t}\right)$, where $V_{t}$ and $E_{t}$ represent the current node and edge sets, respectively. It also includes the dynamically generated node embeddings $h_{v}$, which capture both local and global structural features. The state can be represented as:

(3) $$S_{t}=\left\{h_{v},\left.G_{t}\right\},\;v\in V_{t}\right.$$

These embeddings are updated at each step, allowing the agent to adapt to changes in graph structure and make informed decisions. 

The action $A_{t}$ refers to selecting a node $v\;\in \;V_{t}$ for inclusion in the subgraph. This action is determined by evaluating *Q*-values using a Deep *Q*-Network (DQN):

(4) $$v_{t}=\frac{argmaxQ\left(S_{t},u\right)}{u\in V_{t}}$$

where $Q\left(S_{t},\;u\right)$ is the *Q*-value of selecting node u given state $S_{t}$. After selection, the graph is updated to $G_{t+1}$, and the process repeats until the subgraph satisfies the required size constraint. In practice, we maintain an action mask *m_t_*(*u*) ∈ {0, 1} to exclude invalid candidates, including (i) nodes that have been removed from the graph at previous steps, (ii) nodes that violate hard constraints (if any), and (iii) padded dummy nodes introduced by mini-batching. The masked action selection is implemented as

(5) $$v_{t}=\arg\begin{array}{c} max\\ u\epsilon V_{t} \end{array}\left(Q_{\theta }\left(S_{t},u\right)+\left(1-m_{t}\left(u\right)\right)\cdot \left(-\infty \right)\right)$$

so that only valid candidates participate in the maximization.

Optional candidate pruning. For scalability on larger graphs, RL-SGF can optionally evaluate the *Q*-scorer only on a reduced candidate set *C_t_* ⊆ *V_t_* (e.g., heuristic pre-filtering by degree/centrality, or top-M preselection by a lightweight score). In this case, Equation (5) is applied on *C_t_* instead of *V_t_*, trading a controllable amount of optimality for improved runtime.

Mini-batch training with variable sizes. To enable mini-batch training across graphs (or intermediate states) with different numbers of nodes, we pad node lists within a batch to *N_max_* and apply the corresponding mask *m_t_*(*·*). All maximization operations (e.g., *max_u_ Q_θ_* (*S_t_*, *u*)) are computed only over unmasked positions, ensuring correctness under padding.

#### 4.2.2. PF Metric for SGF in RL-SGF

In RL-SGF, the Potential Friends (*PF*) metric serves as the core sparse evaluation function during DRL optimization. The agent seeks to maximize cumulative rewards, which are inversely related to the PF value of the resulting subgraph.

The *PF* metric extends the traditional *k*-triangle and *k*-line metrics by capturing latent connectivity patterns. For any two nodes u and v in a subgraph *T*, define ${CN}_{k}\left(u,v\right)$ as the set of *k*-hop common neighbors, including u and v themselves. Let *G* = (*V*, *E*) be an undirected graph. For two nodes u,v∈V, we define the set of *k*-hop common neighbors as

(6) $${CN}_{k}\left(u,v\right)=\left\{\omega \epsilon V\left|dist\left(\omega ,u\right)\leq k\;and\;dist\left(\omega ,u\right)\leq k\right.\right\}$$

where *dist*(*·*,*·*) denotes the shortest-path distance in *G*.

Intuitively, ${CN}_{k}\left(u,v\right)$ characterizes the latent structural proximity between nodes u and v by capturing both direct and indirect interactions within a *k*-hop neighborhood. When $\left(u,v\right)\in E$, the direct edge is treated as an explicit interaction and is incorporated into the *PF* computation accordingly. Then:

(7) $${PF}_{T}\left(u,v\right)=\left\{\begin{array}{l} \left\{{CN}_{k}\left(u,v\right)\right\}\;\;\;\;if\;v\notin N_{k}\left(u\right)\\ \left\{{CN}_{k}\left(u,v\right)\cup \left(u,v\right)\right\}\;if\;v\in N_{k}\left(u\right) \end{array}\right\}$$

The total *PF* value of subgraph *T* aggregates across all node pairs:

(8) $${PF}_{T}={⋃}_{\left(u,v\right)\in V_{T}}{PF}_{T}\left(u,v\right)$$

#### 4.2.3. Reward Definition in RL-SGF

In the RL-SGF model, the reward at each time step *t* is designed to guide nodes election that minimizes the *PF* value of the remaining subgraph. Let $R_{t}$ denote the reward at time step *t*, and let *F* be the set of nodes currently included in the feasible solution. The reward is defined as:

(9) $$R_{t}=-PE\left(G\backslash \left\{v_{1},v_{2},\ldots ,v_{y}\right\}\right)$$

where $PF\left(G\backslash \left\{v_{1},v_{2},\ldots ,v_{t}\right\}\right)$ represents the *PF* value of the remaining subgraph after nodes $v_{1}$ through $v_{t}$ have been removed. The goal is to maximize the cumulative reward over all *k* selections:

(10) $$max\sum_{t=1}^{k}R_{t}=max\sum_{t=1}^{k}\left(-PE\left(G\backslash \left\{v_{1},v_{2},\ldots ,v_{t}\right\}\right)\right)$$

Formula Explanation

- $R_{t}$: The reward received at time step *t*, negatively proportional to the *PF* value of the residual graph.
- $PF\left(G\;\backslash \left\{v_{1},\ldots ,v_{t}\right\}\right)$: The PF metric of the graph after removing selected nodes.
- Objective: Maximizing cumulative reward encourages the agent to generate subgraphs with minimal social interaction (high sparsity).

Step-wise Reward Formulation.

RL-SGF adopts a step-wise reward mechanism rather than assigning a terminal reward only. After each node selection, the remaining graph $G_{t}\;$is evaluated and the reward is computed as

$$R_{t}=-PF\left(G_{t}\right)$$

which stabilizes gradient updates and significantly accelerates convergence during training.

Choice of Discount Factor.

The discount factor is set to *γ* = 0.95. Since SGF is an accumulated long-horizon decision-making problem, the sparsity of the final subgraph is influenced by multiple sequential actions. A relatively large *γ* emphasizes long-term effects. We conducted controlled experiments with *γ* ∈ {0.8, 0.9, 0.95, 0.99}, and *γ* = 0.95 demonstrated the best stability and performance.

Enforcing the k-node Constraint.

RL-SGF explicitly enforces the final subgraph size requirement |*F*| = *k*. Any action that results in |*F*| > *k* incurs a penalty term

$$R_{penalty}=-C\cdot \left(\left|F\right|-k\right)$$

where the constant *C* is set to 500. This mechanism prevents the policy from violating the node budget while maintaining training stability. Although the SGF task aims to identify a sparse subgraph $V_{T}$ of size *k*, RL-SGF adopts a sequential pruning formulation. At each time step, the agent removes one node from the current graph, and the remaining graph gradually converges to a *k*-node subgraph after |*V*| − *k* steps.

Under this formulation, minimizing the PF value of the remaining graph is equivalent to minimizing the PF of the final selected subgraph. Nodes that contribute heavily to the PF metric are preferentially removed during the pruning process, which effectively eliminates highly connected or structurally redundant nodes. As a result, the remaining node set forms a subgraph whose internal connectivity is minimized under the PF metric, thereby aligning the step-wise reward design with the original SGF objective. An alternative reward design is to define the reward directly based on the PF value of the constructed subgraph $PF\left(V_{T}\right)$. However, such a formulation leads to a sparse and delayed reward signal, which significantly degrades training stability in long-horizon reinforcement learning settings.

The step-wise reward based on the PF of the remaining graph provides dense inter- mediate feedback after each action, enabling faster convergence and more stable policy optimization. Importantly, this design preserves consistency with the SGF objective while substantially improving learning efficiency. This reward function ensures that the reinforcement learning agent is driven to construct sparse subgraphs by sequentially selecting nodes that minimize network cohesion.

#### 4.2.4. Deep Q-Network for SGF in RL-SGF

Based on node embeddings generated by the graph embedding module, the RL-SGF framework uses a Deep Q-Network(DQN) to approximate the action-value function for the SGF task. Compared to traditional reinforcement learning, the DQN design enables learning in continuous state spaces and complex graph environments. This design aligns with attention-based GNN-RL models such as GAPO, which demonstrate that attention mechanisms can improve node-level decision-making in dynamic graph environments [45]. In the DQN architecture, the network is composed of multiple fully connected layers. Specifically, we use 3 fully connected layers, where the first and second layers each contain 128 neurons, and the third layer contains 64 neurons. Each layer uses the ReLU activation function. We clarify that the DQN in RL-SGF does not produce a fixed |*V*|-dimensional output vector. Instead, RL-SGF adopts a shared node-wise *Q*-scorer: for each candidate node *u* ∈ *Vt*, the same *Q*-network *f_θ_*(*·*) outputs a scalar action-value *Q_θ_*(*S_t_*, *u*). Formally, we compute

(11) $$Q_{\theta }\left(S_{t},u\right)=f_{0}\left(\psi S_{t}\right),h_{u}^{\left(I\right)})\epsilon R$$

where *ψ*(*S_t_*) denotes the state representation (including the remaining graph *G_t_* and the dynamic embeddings), and $h_{u}^{\left(I\right)}$ is the embedding of node u at the final layer *I* of the graph embedding module. The scorer *f_θ_*(·) is shared across all candidates and thus naturally supports variable-sized graphs. During decision-making, RL-SGF evaluates Equation (11) over the current candidate set and selects the best node via the node-wise argmax rule in Equation (4), making the network description consistent with the action-selection equation.

The action-value function $Q_{\theta }\left(F,\;v\right)$, parameterized by a learnable set θ, is modeled as:

(12) $$Q_{\theta }\left(F,v\right)=\beta_{1}\cdot ReLU\left(\sum_{u\epsilon N(v)}\beta_{2}\cdot h_{u}^{\left(I\right)}\left\|\beta_{3}\cdot h_{v}^{\left(I\right)}\right.\right)$$

Formula Explanation

- $\beta_{1}$: Scales the ReLU activation output.
- $\beta_{2}$: Weights the embeddings of neighbors $h_{u}^{\left(I\right)}$, reflecting local structural information.
- $\beta_{3}$: Encodes global sparse information for the target node *v*.
- $\left\|:\right.$: Refersto “concatenation” (the process of joining), which involves combining the embedding information of the target node with that of its neighboring nodes.

In DQN, the update strategy of the target network is used to enhance the stability of training. The parameters of the target network are updated every fixed number of training steps (for example, every 1000 steps or every 5000 steps). The update method usually employs a soft update strategy, where the parameters of the target network gradually approach those of the main network, rather than completely copying the parameters of the main network at each update. The soft update formula is:

(13) $$\theta_{target}\leftarrow {\tau \theta }_{online}+\left(1-\tau \right)\theta_{target}$$

- *θ_target_*: the parameter of the target network
- *θ_online_*: the parameter of the main network
- *τ*: the soft update factor (usually set to 0.001)

The notation follows that defined in Section 4.1.

Two key techniques enhance the DQN’s stability and performance: a twin network architecture and experience replay. The twin architecture includes:

- A policy network that generates *Q*-values for current decisions.
- A target network that is periodically updated from the policy network to stabilize learning.

**Figure 1:**
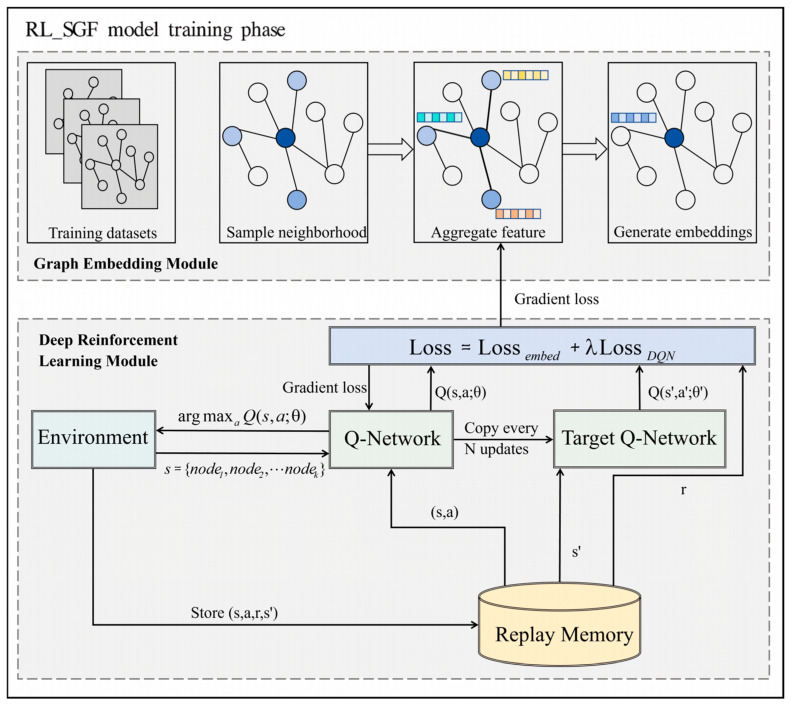
The framework of the RL-SGF model. The Graph Embedding Module (**top**) transforms the input graph into node embeddings that incorporate sparse information. The Deep Reinforcement Learning Module (**bottom**) optimizes the selection of nodes based on the Q-value, using a twin network architecture and experience replay to enhance stability and performance.

As shown in Figure 1, the *Q*-network and its target counterpart collaboratively evaluate candidate nodes’ sparsity (*Q*-value). The use of experience replay further stabilizes training by decoupling consecutive samples, allowing the agent to generalize across various graph structures while reducing overfitting.

### 4.3. MDP Definition

To make the decision-making process of RL-SGF more explicit, we provide a complete definition of the Markov Decision Process (MDP) adopted in this work. The MDP is formulated as a tuple.

(14) $$M=\left(S,\;A,T,R,\gamma \right)$$

- State Space *S*: Each state *S_t_* contains three components:

$$S_{t}=\left(G_{t},F_{t},H_{t}\right)$$

where *G_t_* = (*V_t_*, *E_t_*) is the remaining graph at step *t*, *F_t_* is the set of already selected nodes (|*F_t_*| ≤ *k*), and *H_t_* = {*h_v_* | *v* ∈ *V_t_*} denotes the dynamic embeddings generated by the joint graph encoder.

- Action Space *A*: An action *At* ∈ *A* selects a node from the remaining node set:

*A_t_* = *v_t_*, *v_t_* ∈ *V_t_*

The selected node is appended to the subgraph:

*F*_*t*+1_ = *F_t_* ∪ {*v_t_*}

and any violation of |*F*_*t*+1_| = *k* triggers a penalty in the reward.

- State Transition Function T: Given current state *S_t_* and action *A_t_* = *v_t_*, the transition is defined as:

*G*_*t*+1_ = (*V*_*t*+1_, *E*_*t*+1_), *V*_*t*+1_ = *V_t_*\{*v_t_*}

*E*_*t*+1_ = {(*i*,*j*) ∈ *E_t_*|*i*,*j* ∈ *V*_*t*+1_}

After the graph is updated, embeddings are recalculated:

*H*_*t*+1_ = *Encoder*(*G*_*t*+1_)

Although the environment changes dynamically due to re-embedding, the transition satisfies the Markov property because *S_t_*_+1_ depends solely on (*S_t_*, *A_t_*).

- Discount Factor γ: The discount factor is set to

*γ* = 0.95,

which encourages the agent to consider the long-term sparsity reward rather than focusing on immediate PF reduction. Experiments demonstrate that *γ* ∈ [0.90, 0.99] maintains stable learning behavior, with *γ* = 0.95 yielding the best balance between exploration depth and optimization stability.

This complete MDP specification ensures a rigorous formalization of RL-SGF, supporting a clearer understanding of its optimization process. Due to the NP-hard nature of the SGF problem, RL-SGF does not guarantee a globally optimal solution. Instead, the learned policy converges to a high-quality approximate solution, which consistently achieves lower PF values than existing baseline methods in empirical evaluations. This behavior is consistent with the standard practice in reinforcement learning for combinatorial optimization problems, For the definitions and implications of the parameters involved in the formulas, please refer to Table 1.

### 4.4. Joint Optimization Strategy

To tightly couple the graph embedding module with the deep reinforcement learning module and overcome the mismatch issues in traditional two-stage approaches, we propose a joint optimization strategy. This design reduces error propagation between module and enables end-to-end training of the entire RL-SGF framework, thereby improving performance and task relevance.

The overall loss function is defined as:

(15) $$L=L_{embed}+\lambda \cdot L_{DQN}$$

where *λ* is a hyperparameter balancing the contributions of the embedding loss and the reinforcement learning loss. By optimizing this joint objective, RL-SGF produces more effective node embeddings that directly enhance subgraph discovery performance.

This unified framework addresses the limitations of traditional methods that treat graph embedding and subgraph optimization as separate stages. This joint optimization philosophy is also reflected in recent cross-domain studies. Wei et al. demonstrate that coupling graph topological embedding with DRL in a unified training framework significantly improves stability and control performance in power-system networks, providing further evidence for the effectiveness of our joint optimization design [37]. Instead, RL-SGF optimizes the sparse score directly in an end-to-end manner, aligning representation learning with task-specific objectives and improving robustness and generalization.

### 4.5. Time Complexity Analysis

#### 4.5.1. Overall Time Complexity

The time complexity of RL-SGF is primarily determined by three stages: node embedding, sparse evaluation, and subgraph construction.

- Node embedding: *O*(|*V*|·|*N*(*v*)|), where |*V*| is the number of nodes and |*N*(*v*)| is the sampled neighborhood size.
- Sparse evaluation: Also *O*(|*V*|·|*N*(*v)*|), due to the reused embeddings and local graph operations.
- Subgraph construction: Selecting top-*k* nodes based on *Q*-values costs *O*(|*V*|). Thus, the overall time complexity is *O*(|*V*|·|*N*(*v*)|), which is efficient and scalable for large graphs.

#### 4.5.2. Comparative Analysis

To highlight the efficiency of RL-SGF, we compare it with two representative baselines: WK and TERA.

The WK algorithm, known for identifying weakly connected components, runs in *O*(|*E*| · *log*|*V*|) time, where |*E*| is the number of edges. While suitable for static and sparse graphs, WK struggles with highly dynamic or dense social networks.

The TERA algorithm, which removes triangles and edges to extract sparse subgraphs, has time complexity $O\left(\delta_{G}^{2}\left|V\right|\right)$, where *δ_G_* is the maximum vertex degree. Though effective in some cases, TERA becomes inefficient for dense graphs, as the number of triangle combinations grows rapidly.

In contrast, RL-SGF avoids explicit enumeration of subgraph candidates by learning from experience. Its embedding-based strategy enables efficient evaluation of node importance, resulting in superior scalability. For large, high-density social networks, the *O*(|*V*|·|*N*(*v*)|) complexity of RL-SGF offers significant computational advantages over WK and TERA.

## 5. Computational Experiments

In this section, we conduct an extensive evaluation of the RL-SGF framework through a series of computational experiments on both real-world and synthetic datasets. We also discuss the process of determining hyperparameters, providing insights into the specific choices made to enhance model performance. Furthermore, we compare the time efficiency of RL-SGF with traditional methods, highlighting the advantages of our approach.

### 5.1. Datasets

#### 5.1.1. Benchmark Datasets

To evaluate the effectiveness of the RL-SGF framework, we utilized six widely recognized benchmark datasets, each reflecting real-world network structures. These datasets include:

- Hvr (Hvr2018): A gene network where nodes represent genes of malaria parasites and edges denote gene interactions. This dataset features highly recombinant characteristics and is commonly used for evaluating community detection algorithms and studying gene functions and evolution.
- Cora (Cora2008): A citation network composed of academic papers as nodes and citation links as edges, with papers categorized into seven classes, often applied in research on paper classification and citation network analysis.
- Citeseer (Citeseer2008): Another citation network similar to Cora, where papers are divided into six categories and represented by high-dimensional binary word vectors, widely used for classification and analysis of high-dimensional data.
- Digg (Digg2009): Asocial media network dataset where nodes represent users and edges represent friendship relations. It includes rich information about user interactions, such as social ties and voting behavior, making it suitable for studying information diffusion, user influence, and social impact prediction.
- Enron (Enron2004): An email communication network from the Enron Corporation, consisting of email users as nodes and communication links as edges, derived from approximately 500,000 emails spanning 1999–2002, frequently used for email network analysis and social network studies.
- Facebook (Facebook2009): A social network dataset where nodes represent users and edges represent friendships. This dataset, which includes user features and anonymized data, is commonly used to analyze social network structures and user behaviors.

Detailed characteristics of these datasets are summarized in Table 2.

#### 5.1.2. Synthetic Datasets

To further test the scalability and robustness of RL-SGF, we generated synthetic datasets using the Barabási–Albertmodel, which captures the scale-free properties typical of many real-world networks [46,47]. These synthetic datasets are divided into two cate- glories: one set with node counts of 1000, 2000, and 5000 to compare RL-SGF against other SGF methods (Table 3), and another set with node counts ranging from 1000 to 100,000 to assess the time efficiency and scalability of the model (Table 4).

### 5.2. Baselines and Evaluation Criteria

To comprehensively evaluate the performance of RL-SGF, we compare it against several baseline methods that are widely recognized in the field of Sparse Subgraph Finding (SGF). The base lines include:

- WK: A traditional SGF method that minimizes the number of *k*-lines with in a subgraph, often used as a standard for evaluating sparse subgraphs [10].
- TERA: An algorithm designed to find sparse groups by leveraging the *k*-triangle metric, which is specifically useful for capturing tightly connected substructures within a network [14].
- GNNM: A graph neural network-based model that incorporates both structural and attribute information to identify subgraphs, providing a modern approach to SGF problems [29].
- GAE (GAT): A variant of the graph autoencoder model that utilizes graph attention networks(GAT) to enhance the learning of node embeddings [48].
- GAE (SGC): Another variant of the graph auto encoder that employs simple graph convolution (SGC) layers, simplifying the model while retaining its capacity to capture subgraph structures [48].

We evaluate the performance of each method using the following metrics, with the sparse parameter *k* set to 1 for fairness. The rationale for this choice is detailed in Section 5.5.3.

- *1*-line: A simplified metric that counts the number of *1*-hop common neighbors between nodes in the subgraph, providing a basic measure of connection [10].
- *1*-triangle: A metric that evaluates the presence of triangles within the subgraph, helping to identify tightly knit substructures [14].
- Potential Friends (PF): The PF value, which measures the sparsity of the subgraph by combining *k*-lines and *k*-triangles to capture both direct and indirect relationships [29].

These criteria provide a comprehensive framework to evaluate the performance of RL-SGF in comparison to the baseline methods, allowing us to assess its ability to effectively 591 identify and optimize sparse subgraphs across different data.

### 5.3. Experimental Design and Configuration 

#### 5.3.1. Hyperparameter Optimization and Tuning

To ensure the optimal performance of the RL-SGF model, we systematically tuned and configured key hyperparameters:

- Learning Rate and Optimizer: The initial learning rate was set to 0.001, with the Adam optimizer selected. To enhance the model’s convergence efficiency, we adopted a dynamic learning rate adjustment strategy during training: a relatively high learning rate was maintained in the early stages to achieve rapid convergence, followed by gradual reductions based on performance on the validation set to prevent overfitting.
- Batch Size: The batch size was set to 64. To identify the optimal value, we conducted a grid search within the range of 32, 64, and 128. We found that a batch size of 64 achieved the best trade-off between computational efficiency and performance.
- Embedding Dimension: The dimension of node embeddings was set to 64. Comparative experiments with embedding dimensions of 32, 64, and 128 demonstrated that a 64-dimensional embedding provided sufficient expressiveness for capturing graph structure information without significantly increasing computational complexity.
- Exploration Strategy: An ϵ-greedy strategy was used to balance exploration and exploitation during node selection. The initial exploration rate (*ϵ*) was set to 0.1 and decayed progressively during training to ensure a smooth transition from early exploration to later exploitation.
- Training Epochs: Each experiment was trained for 200 epochs. To ensure stability in the experimental results, the training process incorporated both a target *Q*-network and an experience replay mechanism, which reduced instability caused by sampling biases and enhanced the model’s generalization ability.

To further evaluate the robustness of RL-SGF with respect to key hyperparameters, a series of sensitivity experiments were conducted by varying the embedding dimension (32, 64, 128, 256), learning rate (1 × 10^−3^ to 5 × 10^−5^), discount factor *γ* (0.90–0.99), and the target-network update interval (100–500 steps). The results indicate that RL-SGF achieves the best and most stable PF performance when the embedding dimension lies in the range of 128–256. Lower dimensions lead to insufficient structural expressiveness, whereas higher dimensions introduce additional computational overhead with negligible performance gain. Learning rate and discount factor variations also show that the algorithm is stable within standard practice ranges, with *γ* = 0.95 providing the most consistent PF reductions. Overall, these observations confirm that RL-SGF demonstrates strong robustness to key hyperparameters.

#### 5.3.2. Experimental Setup and Reproducibility

To ensure the reliability, stability, and reproducibility of the experimental results, the following settings were adopted:

- Independent Runs: All experiments were independently run, with the results in Table 5 and Table 6 coming from 30 independent runs, and the results of the other experiments coming from 5 independent runs. Each run was initialized using a different random seed, chosen from the range [1, 1000], ensuring that the initial conditions for each run were independent. This multi-run setup minimized biases caused by random initialization and provided a more comprehensive evaluation of the model’s performance.
- Hardware Environment: All experiments were conducted on a high-performance machine equipped with an NVIDIA GeForce RTX 3090 GPU, running a Linux operating system. The development environment was based on Python 3.8 and TensorFlow 2.0.
- Impact of Randomness: To eliminate potential biases from random seeds and initial conditions, each independent run of the experiment followed a complete process from training to testing. This rigorous approach ensured that the full experimental workflow was covered in every run.

To ensure statistical reliability, all experiments are independently repeated 30 times. The PF results reported in the tables correspond to the mean and standard deviation over these 30 runs. Furthermore, we compute the 95% confidence interval (CI) for each algorithm on every dataset. Before presenting the main results, we also conduct a paired *t*-test between RL-SGF and the second-best baseline for each benchmark. Across all six datasets, the *p*-values are below 0.01, indicating a statistically significant improvement. The narrow confidence intervals further support the stability of RL-SGF.

### 5.4. Ablation Study

We further conducted ablation studies to examine the contribution of each major component in RL-SGF: (i) removing the joint graph embedding module and replacing it with static node features; (ii) substituting the composite *PF* reward with a single component *k*-line or *k*-triangle reward; and (iii) disabling the action-pruning mechanism so that all candidate nodes are evaluated at each step. The results show that removing the joint embedding significantly weakens the *PF* minimization capability, single sparsity indicators consistently underperform compared to the composite *PF* reward, and disabling action pruning causes slower convergence and unstable *PF* performance. These findings collectively demonstrate that the joint embedding, composite *PF* reward, and action-pruning strategy are all essential to the effectiveness of RL-SGF.

### 5.5. Main Results

**Figure 2:**
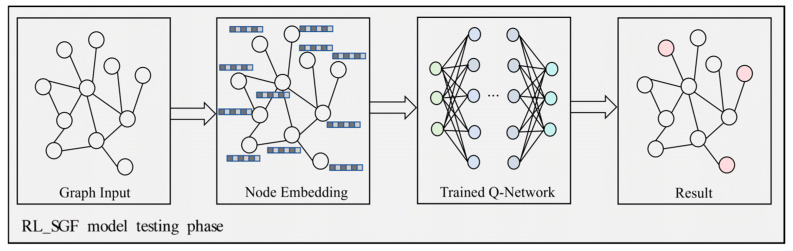
The testing phase of the RL-SGF model. The process begins with the input graph, followed by node embedding generation. The embeddings are then fed into the trained *Q*–network, which predicts the optimal sparse subgraph, highlighted in the result phase. Different node colors indicate different states in the RL-SGF testing phase: uncolored nodes represent original graph nodes, colored nodes in the embedding and *Q*-network stages denote learned node representations, and highlighted nodes in the result indicate selected nodes.

As shown in Figure 2, during the testing phase, the RL-SGF model processes the input graph to generate node embeddings, which are then utilized by the trained *Q*-network to identify the optimal subgraph configuration. This process ensures that the model’s predictions are based on the learned graph representations an optimized decision-making framework.

#### 5.5.1. Same Subgraph Constraint

In the experiments conducted on six benchmark datasets and synthetic datasets (Table 5 and Table 6), we performed a comprehensive comparison of the performance of different algorithms. It is important to note that the WK algorithm, due to its limitations, was excluded from the core comparative results. This algorithm is only applicable to simple and sparse network structures and fails to capture weak connections effectively within complex networks. Specifically, the WK algorithm yields zero or extremely low values for metrics such as *PF*, *1*-line, and 1-triangle, and is incapable of constructing meaningful weak connection subgraphs. While the WK algorithm may have certain applicability in small-scale or simple networks, its performance is inferior to that of other algorithms in large-scale, complex networks. Therefore, it is primarily used as a baseline for validating improvements and superiority of more advanced algorithms.

The experimental results demonstrate that the RL-SGF algorithm performs excellently across all six benchmark datasets and synthetic datasets, particularly showing a remarkable advantage in the PF metric, which is critical for assessing potential weak connections. The PF metric integrates the characteristics of 1-line and 1-triangle, providing a comprehensive description of the potential relationships between nodes within the graph. RL-SGF’s optimization capability in this metric highlights its unique strengths and technological advancements in the task of weak connection subgraph mining.

From the bench mark dataset results (Table 5), it is clear that RL-SGF performs optimally in terms of the PF metric on nearly all datasets. Specifically, RL-SGF, by integrating deep reinforcement learning with an optimization framework for graph embedding, is able to effectively balance weak connectivity with overall connectivity, successfully identifying more fragile subgraphs in high sparsity networks such as Cora and Citeseer. For example, in the Citeseer dataset, RL-SGF achieved a *PF* value of 333, significantly lower than the second-best algorithm’s value of 1998. Even in larger datasets like Enron, which features a large email communication network with tightly connected clusters of nodes and sparse inter-cluster connections, RL-SGF excels in identifying and optimizing weak connections, outpacing the second-best algorithm (RL-SGF = 47,789, second-best algorithm = 6,171,112).

In high-density networks such as Facebook and Digg, as network size increases, weak connections become more evenly distributed and harder to distinguish. In these datasets, RL-SGF achieved *PF* values of 125,887 and 39,567, respectively. Although the gap with the second-best algorithm or the best algorithm is smaller, RL-SGF still demonstrates its effectiveness and competitiveness in dealing with complex social networks. This suggests that even when faced with higher challenges, RL-SGF remains capable of delivering optimized weak connection mining results.

Moreover, RL-SGF has shown significant abilities in reducing redundant paths and triangle closures. In nearly all datasets, RL-SGF outperformed other algorithms on both the 1-line and 1-triangle metrics, further proving its excellence in optimizing weak connections within high-density networks.

In terms of standard deviation, RL-SGF demonstrated greater stability across the six benchmark datasets compared to other algorithms. Notably, on datasets such as Hvr, Cora, and Citeseer, RL-SGF exhibited a smaller standard deviation, indicating higher consistency in performance across different random seeds. While the standard deviation increased in larger datasets like Digg, Enron, and Facebook, this increase was observed across all algorithms, indicating that as dataset size grows, the complexity of the network introduces some instability to all algorithms. In comparison, other algorithms, such as GAE (GAT), exhibited larger standard deviations, particularly in Enron and Facebook datasets, where the standard deviations were ±935,817 and ±27,906, respectively. This may be due to the higher sensitivity of these algorithms to initial conditions, resulting in less stable outcomes.

In experiments on synthetic datasets (Table 6), RL-SGF demonstrated its scalability and robustness. As the number of nodes increased from 1000 to 5000, RL-SGF consistently outperformed other algorithms in the PF metric, with particularly strong performance in high-density networks, where both the *1*-line and *1*-triangle values remained highly competitive. This indicates that RL-SGF, through its reinforcement learning strategy, is capable of constructing the most sparse subgraphs, whereas traditional heuristic methods often fail to maintain such performance as network sizes increase.

Additionally, RL-SGF displayed exceptional consistency in terms of standard deviation. In particular, on the Synthetic_1000 dataset, RL-SGF’s PF value standard deviation was ±0, demonstrating its stability when processing small-scale synthetic networks, unaffected by randomness. In contrast, TERA showed a standard deviation of ±7 on the same dataset, exhibiting some level of fluctuation. As the dataset size increased, RL-SGF maintained a low standard deviation in the Synthetic_2000 and Synthetic_5000 datasets, further confirming its reliability and stability in medium and large networks.

In conclusion, RL-SGF not only excels in the PF metric but also exhibits excellent adaptability and scalability across networks of various sizes and densities. Its advantages stem from the integration of an optimization strategy and reinforcement learning framework, which allows dynamic adjustment of embeddings and reward mechanisms, directly optimizing weak connectivity and resolving the misalignment between embedding learning and structural optimization that traditional algorithms struggle with. This makes RL-SGF a highly accurate and stable solution for large-scale, complex networks.

#### 5.5.2. Different Subgraph Constraints

**Figure 3:**
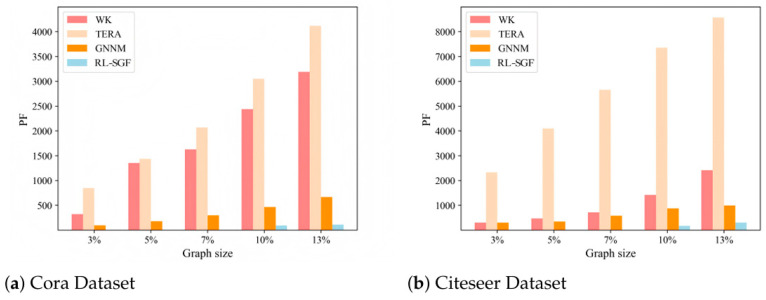
Model performance comparison (*PF* values) of different algorithms as the size of generated sparse subgraphs varies from small to large.

We compared the variation in PF values of different algorithms for solving sparse subgraphs of varying sizes. Using the Cora and Citeseer datasets as examples, we analyzed the performance by generating sparse subgraphs with sizes corresponding to 3%, 5%, 7%, 10%, and 13% of the original graph. Figure 3a,b show the experimental results for the Cora and Citeseer datasets, respectively. The *x*-axis represents the size of the sparse subgraph. From these figures, it is evident that the RL-SGF algorithm proposed in this chapter has a clear advantage. As the size of the subgraph increases, the advantage of the RL-SGF algorithm becomes more pronounced. The *PF* values of the WK and TERA baseline algorithms increase rapidly, especially on the Citeseer dataset, where the *PF* value of the TERA algorithm is significantly higher than the other three algorithms. Analysis of the Citeseer network reveals that the high *PF* values are primarily due to the relatively dense connections between certain nodes in the network, which introduces a large number of *PF* values.

#### 5.5.3. Impact of k on Performance Analysis

**Figure 4:**
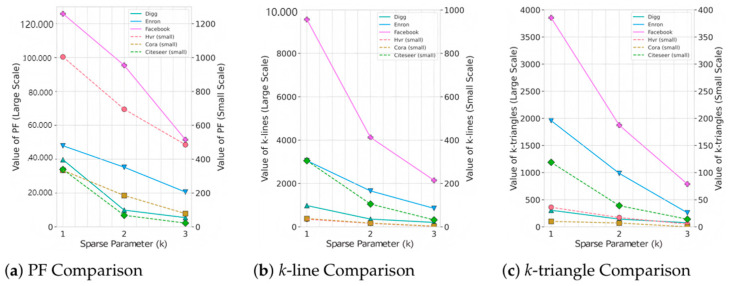
Performance of *PF* value, *k*-lines and *k*-triangles with varying *k* across six datasets under RL-SGF.

In this study, we introduce *k*-line, *k*-triangle, and *PF* metric to evaluate subgraph sparsity, where *k* is a critical parameter. To clarify its impact, we conducted experiments on six datasets: Hvr, Cora, Citeseer, Enron, Digg, and Facebook. The results are presented in Figure 4.

- *PF* Metric Figure 4a: The *PF* metric integrates *k*-line and *k*-triangle to provide a com- prehensive evaluation of subgraph sparsity. As *k* increases, the *PF* value consistently decreases because chain connections and triangle structures are progressively eliminated. This trend is more significant in large datasets, while smaller datasets show a more gradual decline. This demonstrates that the PF metric effectively captures weak connections and sparsity, with *k* directly influencing its measurement scope and accuracy.
- *k*-Line Metric Figure 4b: The k-line metric measures chain-like connections, defined as node pairs with a shortest path length less than *k*. As *k* increases, the number of *k*-lines decreases due to the inclusion of longer paths. This reduction is more pronounced in large-scale datasets (e.g., Facebook and Enron), where chain connections drop significantly at *k* = 3. In smaller datasets (e.g., Cora and Hvr), the changes are less noticeable due to the limited graph size.
- *k*-Triangle Metric Figure 4c: The k-triangle metric measures local triangle structures where all node pairs in a triplet have a shortest path length less than *k*. Increasing *k* significantly reduces the number of triangles, indicating a decrease in local dense structures. This trend is particularly evident in large-scale graphs like Facebook and Enron, validating the effectiveness of *k*-triangle in assessing sparsity.

As derived from the preceding analysis, employing a small neighborhood size *k* enables efficient capture of local structural features within the graph while preserving low computational overhead. The optimal configuration is established at *k* = 1, which facilitates precise extraction of local graph structures by focusing exclusively on the immediate neighbors of each node. This approach minimizes computational expense, eliminates redundant features and unnecessary computations, and consequently attains an optimal performance-efficiency trade-off on large-scale datasets.

Conversely, larger *k* values (*k* ≥ 2), while capable of incorporating broader structural information, incur a computational complexity that escalates exponentially with *k*. In large-scale graph applications, this leads to substantially diminished efficiency. Furthermore, the marginal performance gains progressively diminish, and the additional structural information fails to compensate for the heightened computational burden. Consequently, selecting *k* = 1 effectively manages computational resources while maintaining model efficacy, rendering it particularly well-suited for processing requirements in large-scale graph datasets.

### 5.6. Model Analysis 

#### 5.6.1. Time Efficiency and Scalability

**Figure 5:**
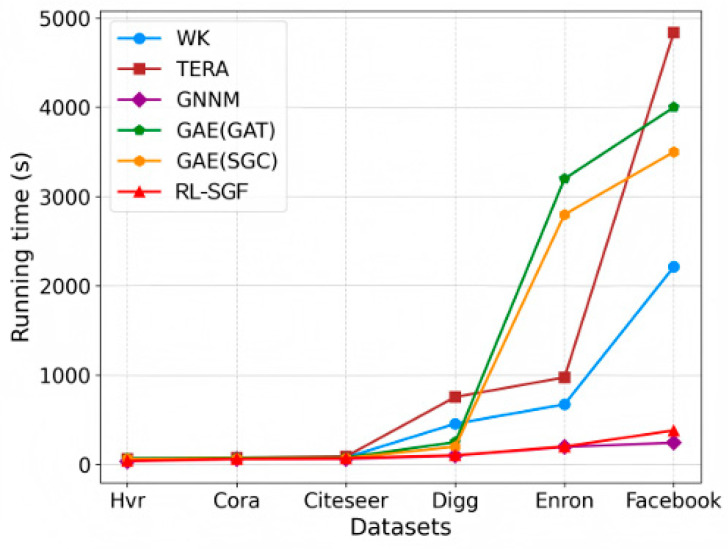
Time efficiency and scalability studies of different algorithms on benchmark datasets.

To evaluate the time efficiency and scalability of the RL-SGF algorithm, we compared the running times of six algorithms across several real-world datasets. The experimental results are shown in Figure 5. Based on these results, the following conclusions can be drawn: 796.

As the dataset size increases (from Hvr to Facebook), the running time of all algorithms increases. However, the growth rate of RL-SGF is significantly lower than that of the other algorithms, especially in large-scale networks where it performs notably well. In small-scale datasets (e.g., Hvr and Cora), the running time differences among the algorithms are minimal, and RL-SGF’s performance is comparable to that of other algorithms, indicating that its efficiency advantage is not significant in smaller datasets. However, as the dataset size increases, the efficiency advantage of RL-SGF becomes more evident. On medium-scale datasets (e.g., Digg), RL-SGF significantly reduces running time by optimizing redundant search space.

The differences in algorithm efficiency are especially prominent in high-density net- works (e.g., Enron and Facebook). The TERA algorithm relies on shortest path and triangle count calculations, and its computational complexity increases significantly when handling high-density or large-scale networks. In contrast, RL-SGF dynamically optimizes node selection through a reinforcement learning framework. By using a Q-network to select the optimal node based on the current graph state, RL-SGF avoids the need for exhaustive searches across the entire graph, preventing the exponential growth of the search space and thereby significantly reducing the search space, which results in higher efficiency in terms of running time. A similar observation is reported in GAPO, where graph-attention-based RL significantly reduces the search space for multi-hop task offloading, reinforcing the efficiency advantage of RL-driven graph optimization [45]. Furthermore, the time efficiency improvement of RL-SGF is attributed to its end-to-end optimization framework that combines reinforcement learning with graph embedding techniques. Through this joint optimization approach, RL-SGF reduces redundant computations when dynamically optimizing subgraph structures, significantly lowering time complexity.

Although GNNM performs slightly better in terms of computational time on the Facebook dataset, which may be due to its advantage in encoding local features, RL-SGF demonstrates superior adaptability in ultra-large, high-density networks. This is due to its dynamic optimization capabilities, which enable it to handle sparse connections in a more effective and scalable manner.

#### 5.6.2. Visual Ana

**Figure 6:**
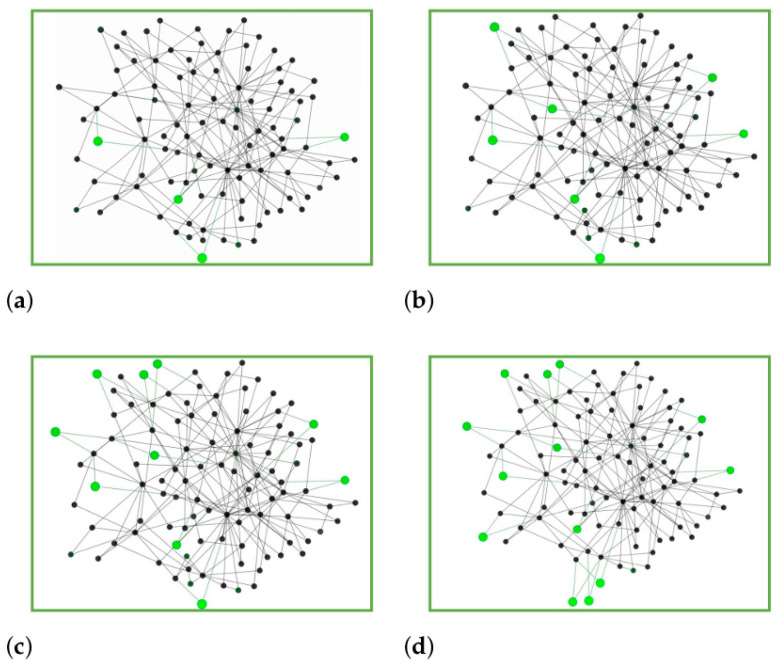
A simple example illustrates how RL-SGF works. (**a**–**d**), respectively, showcase the discovery of sparse subgraphs of sizes 4, 7, 10, and 13, with the sparse subgraph solutions composed of green nodes.

To further demonstrate the effectiveness of RL-SGF in sensor network applications, we visualized sparse subsystems of varying sizes (4, 7, 10, and 13 sensor nodes) within the same sensor network topology, as shown in Figure 6 Using the *PF* metric, we evaluated the sparsity of each sensor subsystem, resulting in *PF* values of 0, 0, 3, and 3, respectively. These visualizations illustrate RL-SGF’s capability to consistently identify optimal sparse subsystems in sensor networks, where minimizing connectivity while maintaining coverage is crucial for energy efficiency and fault tolerance. The sensor network case study demonstrates that RL-SGF can effectively identify subsystems with minimal communication overhead, which is particularly valuable in resource-constrained sensor network environments. The green nodes in the visualization represent the selected sensor nodes that form the sparse subsystem, while the gray nodes represent the remaining network infrastructure. This spatial distribution analysis confirms that RL-SGF successfully identifies sensor configurations that minimize redundant connections while maintaining essential coverage, reinforcing the findings from our quantitative analysis.

In practical sensor network deployments, such sparse subsystem discovery enables efficient cluster formation, reduces communication congestion, and enhances network lifetime by identifying minimally connected yet functionally complete sensor groups. The progressive expansion from 4 to 13 nodes in our visualization demonstrates RL-SGF’s scalability in handling growing sensor network sizes while maintaining optimal sparsity characteristics.

### 5.7. Case Study: Sensor Network Analysis on a Real Deployment Dataset

To provide concrete evidence for the effectiveness of sparse subgraph selection in sensor-network scenarios, we conduct a case study based on a real-world sensor deployment dataset. All experiments are carried out in a controlled simulation-based setting on a communication graph constructed from the deployment data.

#### 5.7.1. Dataset and Network Construction

We use the Intel Berkeley Research Lab Sensor Network Dataset, which contains measurements from *N* = 54 sensors deployed in an indoor environment. Each sensor corresponds to a node in the network.

To construct the communication topology, we build an undirected graph where an edge (*i*, *j*) exists if the Euclidean distance between sensors *i* and *j* is below a predefined threshold *r*:

(16) $$\|p_{i}-p_{j}{\|}_{2}\leq r$$

This procedure results in a connected graph that reflects realistic spatial constraints of sensor communication. All subsequent evaluations are performed on this constructed graph, and no assumption is made about online deployment or real-time execution.

#### 5.7.2. Baselines

We compare RL-SGF with the following baseline methods:

- Original: the full communication graph without any node removal.
- Random-*k*: randomly selecting *k* nodes and taking the induced subgraph (results averaged over 20 random trials).
- Degree-based pruning: selecting the top-*k* nodes with the highest degrees.
- RL-SGF(ours): the proposed reinforcement learning based sparse subgraph finder.

All methods are constrained to produce subgraphs with the same node budget *k* to ensure a fair comparison.

#### 5.7.3. Evaluation Metrics for Sensor Network Case Study

We evaluate the selected subsystems from two complementary perspectives

Communication efficiency.

Communication efficiency is measured by the average shortest-path length (*ASPL*) within the selected subgraph *Gs* = (*Vs*, *Es*):

(17) $$ASPL(G_{S})=\frac{1}{|V_{S}|\left(|V_{S}|-1\right)}\sum_{i\neq j\in V_{S}}{dist}_{Gs}(i,j)$$

where *distGs* (·) denotes the hop distance between two nodes in *Gs*.

Robustness under node failures.

To evaluate robustness, we emulate single-node failures by removing one node at a time from *G_s_*. We define the robustness score as the fraction of cases in which the remaining graph remains connected:

(18) $$Robustness(G_{s})-\frac{1}{\left|V_{s}\right|}\sum_{i\in V_{s}}\mathrm{II}\left(C(G_{s}\backslash \{v\})-1\right)$$

where *C*(·) denotes the number of connected components and *I*(·) is the indicator function.

#### 5.7.4. Results and Discussion

Table 7 summarizes the experimental results. Compared with random selection and simple degree-based pruning, RL-SGF consistently yields subgraphs with shorter average path lengths, indicating improved communication efficiency. Moreover, the selected subgraphs exhibit higher robustness under emulated single-node failures, suggesting that RL-SGF preserves structurally important nodes more effectively.

These results demonstrate that, even when evaluated on a communication graph derived from a real sensor deployment, RL-SGF can identify compact subsystems that maintain efficient communication paths and strong structural robustness.

It is important to note that the above results are obtained under a controlled, simulation-based evaluation on a communication graph constructed from a real sensor deployment dataset. Rather than serving as a definitive assessment of real-world performance, this case study provides empirical evidence that RL-SGF is capable of identifying compact subgraphs that preserve favorable structural properties, such as shorter communication paths and higher robustness to single-node failures, in a realistic yet simplified setting.

We emphasize that the sensor network considered here is of relatively small scale and static topology, and thus does not capture all complexities of practical sensor-network deployments, such as large-scale dynamics, time-varying connectivity, or communication noise. Nevertheless, the observed trends suggest that learning-based sparse subgraph selection has the potential to complement heuristic approaches when designing or analyzing sensor-network subsystems. A more comprehensive evaluation on larger and dynamic sensor networks is left as an important direction for future work.

## 6. Conclusions

This paper introduces the RL-SGF framework, which successfully applies deep reinforcement learning to the problem of Sparse Subgraph Finding (SGF), addressing the limitations of traditional methods in large, complex sensor network systems. By integrating embedding learning with sparse subsystem optimization within a unified framework and directly optimizing the selection strategy for sparse graphs through a unified loss function, RL-SGF ensures efficient coordination between embedding representation and sparse sub-system discovery, thereby enhancing the model’s effectiveness and robustness in sensor network applications. Experimental results demonstrate that RL-SGF excels across various benchmark and synthetic datasets, particularly in capturing subsystem sparsity using the *PF* metric. Additionally, time efficiency analysis confirms the scalability of RL-SGF, highlighting its advantages in handling large-scale sensor networks.

Our findings align with the broader research trend summarized by Tam et al., who emphasize the importance of combining GNNs and DRL in next-generation intelligent network systems [36]. The RL-SGF framework not only provides an efficient solution for identifying sparse subgraphs but also opens new avenues for applying graph mining techniques to intelligent sensor network systems. By modeling complex sensor networks as graphs and leveraging advanced machine learning methods, RL-SGF facilitates the discovery of sparse subsystems that are critical for system reliability, resource optimization, and anomaly detection. Future work will focus on extending RL-SGF to dynamic sensor networks and integrating real-time data streams for adaptive sparse subsystem discovery.

Despite the strong empirical performance of RL-SGF, several limitations remain. (1) The model requires repeated embedding computations, resulting in higher training cost compared with lightweight heuristics. (2) Although the *PF* metric integrates both *k*-line and *k*-triangle structures, its sensitivity to different topologies still depends on dataset-specific hyperparameters. (3) The proposed method is designed for static graphs and cannot be directly applied to dynamic or streaming networks. (4) The computational efficiency of RL-SGF becomes increasingly critical when scaling to sensor networks with several thousand nodes. The current framework involves repeated joint-embedding updates and sequential decision steps, which introduce non-trivial computational overhead.

Future research will focus on developing an incremental or dynamic version of RL-SGF, incorporating more efficient approximate embedding techniques to further reduce training overhead, designing context-aware adaptive *PF* weighting to improve generalization across heterogeneous network structures, and introducing lightweight graph encoders and parallelized policy-learning mechanisms to enhance the scalability of RL-SGF on large-scale real-world sensor networks.

## Figures and Tables

**Table 1:** Notation used in the MDP definition of RL-SGF.

Symbol	Description
*G_t_*	Remaining graph at step *t*
*V_t_*	Node set of *G_t_*
*E_t_*	Edge set of *G_t_*
*F_t_*	Selected node set at step *t* (|*F_t_*| ≤ *k*)
*H_t_* = {*v*}	Node embeddings at step *t*
*h_v_*	Embedding of node *v*
*A_t_* = *v_t_*	Action selecting node *v_t_*
*S_t_*	MDP state at step *t*
*T*	State transition function
*γ*	Discount factor
*k*	Required SGF subgraph size

**Table 2:** Benchmark Datasets.

Dataset	|*V*|	|*E*|	Avg. Degree
Hvr	304	3263	21.467
Cora	2708	5429	4.009
Citeseer	3264	4532	2.777
Digg	29,652	84,781	5.718
Enron	36,692	183,831	10.020
Facebook	63,392	816,831	25.771

**Table 3:** Synthetic Datasets for Model Performance Comparison.

Dataset	|*V*|	|*E*|	Avg. Degree
Synthetic_1000	1000	1373	2.746
Synthetic_2000	2000	2341	2.341
Synthetic_5000	5000	6875	2.750

**Table 4:** Synthetic Datasets for Time Efficiency and Scalability Comparison.

Dataset	|*V*|	|*E*|	Avg. Degree
Synthetic_1000	1000	1373	2.746
Synthetic_5000	5000	6875	2.750
Synthetic_10,000	10,000	16,757	3.351
Synthetic_50,000	50,000	69,104	2.764
Synthetic_100,000	100,000	168,206	3.360

**Table 5:** Results on benchmark datasets.

Dataset	Metrics	WK	TERA	GNNM	GAE(GAT)	GAE(SGC)	RL-SGF
Hvr	PF	48 ± 2	12,699 ± 641	2023 ± 105	1853 ± 138	2382 ± 98	**1009** **± 58**
	1-line	0 ± 0	324 ± 9	68 ± 2	41 ± 2	55 ± 2	**32** **± 1**
	1-triangle	0 ± 0	2342 ± 165	39 ± 3	45 ± 3	48 ± 3	**34** **± 2**
Cora	PF	2475 ± 121	3112 ± 205	1998 ± 151	2467 ± 260	2782 ± 162	**333** **± 29**
	1-line	0 ± 0	348 ± 8	211 ± 5	230 ± 10	234 ± 5	**39** **± 1**
	1-triangle	0 ± 0	117 ± 9	39 ± 3	46 ± 3	47 ± 3	**11** **± 1**
Citeseer	PF	1377 ± 91	7324 ± 601	5558 ± 476	5456 ± 648	5689 ± 415	**330** **± 30**
	1-line	0 ± 0	789 ± 39	501 ± 26	519 ± 32	589 ± 24	**302** **± 18**
	1-triangle	0 ± 0	504 ± 25	169 ± 9	159 ± 13	202 ± 9	**112** **± 7**
Digg	PF	17,445 ± 156	41,334 ± 915	**39,456** **± 8179**	40,899 ± 685	42,023 ± 2736	39,567 ± 1368
	1-line	0 ± 0	1070 ± 105	**901** **± 98**	924 ± 110	962 ± 83	988 ± 114
	1-triangle	0 ± 0	742 ± 92	509 ± 68	568 ± 85	608 ± 70	**301** **± 40**
Enron	PF	147,345 ± 156	9,648,556 ± 915	6,171,112 ± 754	6,414,102 ± 935,817	6,774,433 ± 685	**47,789** **± 6852**
	1-line	0 ± 0	82,678 ± 6615	50,385 ± 4105	51,441 ± 4408	54,887 ± 4351	**3035** **± 251**
	1-triangle	0 ± 0	567,862 ± 52,145	327,431 ± 30,152	297,356 ± 35,771	395,431 ± 36,211	**1948** **± 202**
Facebook	PF	257,331 ± 2885	197,435 ± 27,486	154,259 ± 23,260	132,886 ± 27,906	146,734 ± 17,405	**125,887** **± 18,985**
	1-line	0 ± 0	9805 ± 682	**8512** **± 625**	9729 ± 931	9963 ± 592	9559 ± 764
	1-triangle	0 ± 0	72,356 ± 6842	67,237 ± 6182	69,349 ± 20,423	74,522 ± 6840	**3849** **± 456**

The best values are in bold, and the second-best are underlined.

**Table 6:** Results on synthetic datasets.

Dataset	Algorithm	*PF*	*1*-line	*1*-triangle
Syn_1000	WK	141 ± 5	2 ± 0	0 ± 0
	TERA	155 ± 7	15 ± 0	11 ± 1
	GNNM	34 ± 2	2 ± 0	7 ± 0
	RL-SGF	7 ± 0	0 ± 0	0 ± 0
Syn_2000	WK	309 ± 18	8 ± 0	0 ± 0
	TERA	381 ± 24	39 ± 2	27 ± 1
	GNNM	43 ± 3	4 ± 0	5 ± 2
	RL-SGF	34 ± 3	3 ± 0	0 ± 0
Syn_5000	WK	273 ± 18	7 ± 0	0 ± 0
	TERA	130 ± 9	14 ± 2	9 ± 1
	GNNM	72 ± 5	8 ± 1	2 ± 0
	RL-SGF	40 ± 3	5 ± 0	0 ± 0

**Table 7:** Case study results on the Intel sensor network dataset (simulation-based).

Method	|*Vs*|	*ASPL* ↓	*Robustness* ↑
Original	54	3.21	0.83
Random-*k*	*k*	4.07	0.60
Degree-based	*k*	3.74	0.72
RL-SGF (ours)	*k*	**3.15**	**0.88**

*ASPL* ↓ indicates that lower values are better, while *Robustness* ↑ indicates that higher values are better. Bold values denote the best performance among all methods.

## Data Availability

No new data were created or analyzed in this study. Data sharing is not applicable to this article.
